# Shedding Light on Shadows: A Cross-Sectional Analysis of Genital Psoriasis and Its Effects on Thai Individuals

**DOI:** 10.1155/2024/7006796

**Published:** 2024-09-09

**Authors:** Prameyuda Watchirakaeyoon, Pantaree Kobkurkul, Kanokvalai Kulthanan, Chayada Chaiyabutr, Chanisada Wongpraparut, Leena Chularojanamontri, Narumol Silpa-Archa

**Affiliations:** Department of Dermatology, Faculty of Medicine Siriraj Hospital Mahidol University, Bangkok 10700, Thailand

## Abstract

**Background:**

Limited knowledge exists about genital psoriasis in Thai individuals.

**Objectives:**

This study aimed to assess the clinical features of genital psoriasis and its effects on quality of life and sexual health in Thai patients.

**Materials and Methods:**

A cross-sectional, self-administered question-based study was conducted at Siriraj Hospital. Participants were psoriasis patients older than 18 years of age with past or current genital involvement. The study assessed quality of life and sexual health.

**Results:**

Among the 50 patients, 33 (66%) were female. The mean (standard deviation) age was 45.4 (±13.4) years. Genital psoriasis was active in 34% of the participants. The mons pubis (48.5%) and labia majora (18.2%) were the most frequently affected sites in females, while in males, the penile shaft (52.9%) and glans (47.1%) were the most common sites. Itch-related symptoms predominated, affecting 82% of patients. The median dermatology life quality index score was 6.5 for females and 10 for males. A sexual health survey revealed that 54% of participants had low self-esteem. This issue was more pronounced in males, with 76.5% reporting lower self-esteem compared to 42.4% of females (*P*=0.022). Males were also more inclined to postpone or avoid marriage (47.1% vs. 15.6%, *P*=0.038) and more embarrassed about sexual activities (63.6% vs. 14.3%, *P*=0.017). In addition, males were more inclined to avoid sexual encounters due to their genital condition. Notably, 42.9% of all patients had never disclosed to a doctor that they had genital psoriasis.

**Conclusions:**

Genital psoriasis impacts quality of life and sexual function, with male patients being particularly impacted. Improved awareness of these issues among health professionals might increase patient satisfaction.

## 1. Introduction

Psoriasis is an immune-mediated inflammatory disease characterized by erythematous, well-defined plaques with silvery scale. This condition is associated with a heightened risk of systemic comorbidities, such as cardiovascular disease, metabolic syndrome, and diabetes. The prevalence of psoriasis varies globally; it affects approximately 2% of the population in the United States [1]. The disease exhibits a bimodal age of onset, with the first peak occurring at approximately 22.5 years and the second peak at age 55 [2]. Psoriasis is more prevalent in adults than in children [3].

Although genital psoriasis can manifest as a single episode in some psoriasis patients, its presence throughout their lifetime affects a notable portion (33% to 63%) of the population [4]. Individuals experiencing genital psoriasis often report itching, pain, burning, and dyspareunia [5, 6]. Aggravators include stress, trauma, certain medications, and friction from sexual activity or tight clothing. Notably, 34% of patients experience worsening of psoriasis after sexual intercourse [7].

The unique skin microbiota and anatomical location of genital psoriasis pose significant therapeutic challenges. Traditional psoriatic treatment modalities may not directly address this specific manifestation. Currently, topical therapy is the conventional treatment approach. For initial therapy, low-potency corticosteroids are recommended. If needed, moderate to potent corticosteroid formulations might be used. Due to the delicate nature of the genital skin, only intermittent and short courses of these more potent steroids are suggested. Other options include topical calcineurin inhibitors and vitamin D analogs [5, 8]. A study by Ryan et al. revealed that ixekizumab was significantly more effective than placebo in treating moderate to severe genital psoriasis, relieving irritation, and enhancing sexual and general well-being [9].

Genital psoriasis profoundly affects patients' sexuality and quality of life [5, 6, 10, 11]. Meeuwis et al. reported a significantly higher mean dermatology life quality index (DLQI) score in patients with genital psoriasis than in those without genital involvement [6]. However, due to the sensitive nature of the condition, both patients and physicians may avoid discussing it. This often leads to suboptimal patient care [10–12]. The likelihood of this happening is particularly true in Asian countries, including Thailand, where cultural sensitivities regarding sexual questions further complicate the issue [13, 14]. The present study aimed to assess the clinical features of genital psoriasis and its effects on quality of life and sexual health in Thai patients.

## 2. Materials and Methods

### 2.1. Study Design and Participants

This cross-sectional, self-administered questions' study was conducted at the Department of Dermatology, Faculty of Medicine Siriraj Hospital, Mahidol University, a tertiary care center in Thailand. The study protocol was approved by the Siriraj Institutional Review Board (approval number Si544/2022). All of the included psoriasis patients had past or current genital involvement and were aged 18 years or older. The data were collected between July 2022 and March 2023. Patients who were illiterate, intellectually disabled, or unwilling to participate were excluded from the analysis.

### 2.2. Sample Size Estimation

The sample size calculation was based on DLQI data from a study by Meeuwis et al. [6]. With an estimated standard deviation of 6.5, an allowable error of 2, alpha of 0.05, and beta of 0.20, and the minimum sample size was 41 patients. After allowing for a 20% margin for missing data, losses to follow-up, and withdrawals, and the final sample size was 50 subjects.

### 2.3. Study Recruitment and Data Collection

Patients enrolled in the study provided written informed consent to participate in this investigation. The questions comprised three sections:

1. Demographics: The demographic information collected included sex, age, nationality, marital status, education level, and alcohol and tobacco consumption patterns.
2. Psoriasis and genital psoriasis: The second section focused on specific details related to the history of psoriasis and genital psoriasis. The investigated areas included the age of disease onset of both conditions, history in involved areas, history of genital lesion symptoms, exacerbating factors, treatment history, and prior disclosures to physicians. Data on the degree of genital involvement were collected using figures that participants labeled themselves.
3. Quality of life and sexual health: General well-being was evaluated using the DLQI questionnaire translated into Thai and validated by Kulthanan et al. in 2004 [15]. In addition, sexual health was assessed through a set of Thai questions formulated by three dermatologists at Siriraj Hospital (N.S., L.C., and C.W.). The questions captured information about sexual behaviors, self-esteem issues, underwear preferences, partner selection, and relationship dynamics in the context of genital psoriasis.

Physicians also documented the patient's body mass index (BMI), psoriasis type, psoriasis area and severity index (PASI) score, and therapeutic interventions.

### 2.4. Statistical Analysis

Statistical analyses were conducted using PASW Statistics, version 18 (SPSS Inc., Chicago, IL, USA). Categorical data are presented as frequencies and percentages. Continuous data are reported as means ± standard deviations or medians with interquartile ranges. For group comparisons, the chi-square test or Fisher's exact test was used for categorical data, while the two-sample *t* test or Mann–Whitney *U* test was used for parametric/nonparametric continuous data. A *P* value ≤ 0.05 indicated statistical significance.

## 3. Results

### 3.1. Baseline Characteristics

This study enrolled 50 patients with genital psoriasis.  Table 1 summarizes the patients' baseline demographics and clinical characteristics. The mean age of the individuals in the cohort was 45.4 (±13.4) years, with females constituting the majority (66%). The mean age at psoriasis onset was 31.6 (±13.1) years, and genital psoriasis manifested at a mean age of 39.5 (±12.2) years. The cohort's mean BMI of 26 (±5.2) kg/m^2^ qualifies as obese according to the World Health Organization and National Institutes of Health guidelines [16]. Regarding marital status, 50% of the patients were married, 34% were single, 10% were divorced, and 6% were separated. Chronic plaque psoriasis dominated, affecting 45 (90%) participants, followed by erythrodermic psoriasis in 5 (10%) patients.

### 3.2. Clinical Characteristics of Patients with Genital Psoriasis

**Figure 1:**
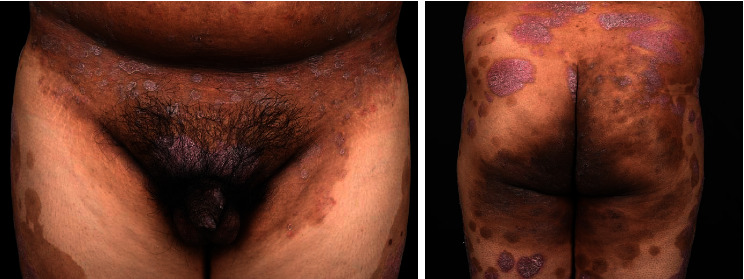
A 30-year-old Thai male with genital psoriasis.

Current genital involvement was noted in 34% (*n* = 17) of the patients (Table 2). Genital psoriasis was the initial presentation in 8% (*n* = 4) of the patients. The previous history of psoriasis included inframammary involvement in 88% (*n* = 44), perianal involvement in 66% (*n* = 33), and both inguinal and axillary involvement in 60% (*n* = 30) of the patients. The most common genital area affected in males was the shaft of the penis (52.9%), followed by the glans (47.1%) and scrotum (29.4%) (Figure 1). In females, the most frequently involved sites were the mons pubis (48.5%), the labia majora (18.2%), the perineum (15.2%), and the labia minora (6.1%). Itch was the leading symptom associated with genital psoriasis (82%), followed by burning and pain (20%) and dyspareunia (4%). Discomfort during intercourse was reported exclusively by male participants. Areas of additional involvement affecting both sexes were the pubic area (44%), buttocks (34%), and inguinal regions (32%). Stress (58%), sleep deprivation (46%), illness (30%), and scratching (30%) were identified as the predominant aggravating factors. Tight clothing, menstruation, and sexual intercourse were cited as aggravating factors in 18%, 12%, and 2%, respectively, of the patients.

### 3.3. Quality of Life, Sexual Health, and Perception of Genital Psoriasis

As shown in Table 3, the median DLQI score among all study participants was 7 (25th percentile = 3 and 75th percentile = 14). Patients with current genital involvement had a median DLQI score of 9 (3.5, 17), which was higher than that of the group without such involvement, whose score was 6 (3, 10.8). A higher median DLQI score of 10 (3, 16) was recorded for male participants than for female participants (6.5 [3, 10]). In response to the specific sexual function question in DLQI question 9 (“Over the last week, how much has your skin caused any sexual difficulties?”), males reported a significantly greater impact on sexual function than females did (odds ratio (OR) = 4.7, *P*=0.016). In addition, males experienced notably lower levels of self-esteem (76.5% vs. 42.4%, OR = 4.4, *P*=0.022) and a greater tendency to postpone or avoid marriage than females did (47.1% vs 15.6%, OR = 4.8, *P*=0.038).

Among the 25 sexually active individuals (42.4% of the females and 64.7% of the males), embarrassment during sexual activities was significantly more common in males (63.6% vs. 14.3%, OR = 10.5, *P* = 0.017). Similarly, men were also more likely to abstain from sexual behavior due to their genital psoriasis than women were, although not to a statistically significant degree (63.6% vs. 35.7%, *P* = 0.165). Interestingly, although male patients were more comfortable with genital examinations by physicians (70.6% vs. 39.4%, OR = 3.7, *P* = 0.037). Only 57.1% had ever discussed their genital psoriasis with their physicians. Furthermore, 60% of the patients, both male and female, indicated that their physicians had neither inquired about nor conducted examinations for genital psoriasis.

### 3.4. Factors Associated with Genital Psoriasis

Subsequent analyses sought to identify factors contributing to the development of genital psoriasis (Table 4). The presence of genital lesions was significantly linked to a higher PASI score than was the absence of genital involvement (7.9 [2.9, 12.6] vs. 4.2 [1.3, 7.8], *P*=0.041). The presence of lesions also correlated with an increased body surface area affected by psoriasis (10% [3%, 15%] vs. 5% [1%, 10%], *P*=0.082). Pubic region involvement strongly indicated genital psoriasis (OR = 5.5, *P*=0.007).

### 3.5. Treatment Modalities for Genital Psoriasis

Table 5 provides a comprehensive overview of the treatment approaches for patients with active genital psoriasis. Among the 17 patients with active genital involvement, a sizeable majority (14 patients, or 82.4%) were receiving treatment. All these patients applied emollients, and most utilized topical therapies, predominantly topical corticosteroids. Six patients (42.9%) were receiving conventional systemic treatments, such as methotrexate, acitretin, and cyclosporine. Two patients (14.3%) received the biologic drug secukinumab, and one patient (7.1%) was treated with narrowband ultraviolet B phototherapy.

Of the 50 patients with past or current genital psoriasis, 58% reported improvement with topical treatments. Emollients were credited with improvements by 36% of patients, while conventional systemic treatments (methotrexate, cyclosporine, and acitretin) were credited with improvements by 30%. Biologic medications, which included IL-17 inhibitors (ixekizumab, secukinumab, and brodalumab) and an IL-23 inhibitor (guselkumab), were associated with improvements in 14% of the patients. Narrowband ultraviolet B phototherapy was reported to be beneficial by 2% of the patients.

## 4. Discussion

Data regarding genital psoriasis in Asian populations are scarce [13, 14]. This study represents one of the most extensive investigations of this manifestation in Thailand. The study population predominantly consisted of female patients, with a median age at disease onset of 39 years for genital psoriasis, typically following the onset of psoriasis. Notably, 8% of the patients presented genital psoriasis as their initial psoriasis manifestation. The median PASI score of 5.1 in our sample indicated mild to moderate disease severity.

The data indicated that 56% of the patients had a BMI of 25 kg/m^2^ or higher; these patients were classified as obese according to the World Health Organization and National Institutes of Health guidelines for Asian populations [16]. This finding aligns with previous studies that identified a relationship between increased BMI and psoriasis severity [17, 18]. Nonetheless, there was no significant difference in the overweight or obese rates between patients with and without active genital psoriasis, consistent with prior findings [17].

The prevalence of current genital psoriasis in our cohort was 34%, echoing the results of earlier studies [7, 17, 19]. A tendency was observed among patients with genital psoriasis to have a history of inverse psoriasis. A significant proportion, between 60% and 88%, had a history of flexural psoriasis involvement. Patients with current genital psoriasis exhibited higher PASI scores and increased involvement of the pubic area. It should be noted that many studies do not differentiate genital psoriasis from inverse psoriasis [8], which may influence the interpretation and comparison of findings across studies.

Itch emerged as the most commonly reported symptom of genital psoriasis in both sexes, consistent with findings from previous studies [7, 10, 12]. Meeuwis et al. [12] also identified itch as one of the most bothersome symptoms, and it was characterized as having the “highest intensity.” Other symptoms, such as burning, discomfort, and dyspareunia, were also identified in our cohort. Our analysis revealed no significant sex differences in the prevalence of genital symptoms (itching, burning, and pain). However, dyspareunia and an exacerbation of disease postcoital activity were exclusively reported by male participants. This finding may be attributed to the frequent occurrence of psoriasis on the penile shaft in males. These observations contrast with the findings of Ryan et al. who suggested that women were more susceptible to experiencing dyspareunia and sexual distress due to genital psoriasis [9].

Stress emerged as the most common aggravating factor for more than half of the participants, aligning with known triggers for psoriasis in general. In addition, tight-fitting clothing and sexual activity were cited as aggravating factors, which can be explained by the Koebner phenomenon, where friction or trauma can induce new psoriatic lesions.

An elevated risk of sexual dysfunction has been associated with psoriasis [20]. A systematic review revealed that the prevalence of sexual dysfunction and erectile dysfunction in these patients ranged from 40% to 55.6% and 34% to 81%, respectively [21]. Anxiety, depression, psoriatic arthritis, and genital psoriasis were identified as factors influencing sexual dysfunction [21]. The presence of proinflammatory cytokines and systemic inflammation in psoriasis may independently lead to sexual or erectile dysfunction. They can affect the levels of sex hormones, cause vascular dysfunctions, or involve other mechanisms that are yet to be fully understood [21]. However, further research is warranted to elucidate these relationships.

Over half of patients with genital psoriasis experience a substantially worse quality of life compared to those with psoriasis on other body areas [11]. Consistent with the findings of previous studies, the median DLQI score for our patients was 7. Although the median score was greater in males than in females, no statistically significant difference was observed. Patients with active genital psoriasis had elevated DLQI scores relative to those without active genital lesions, suggesting a potential improvement in quality of life following lesion resolution [6, 9].

Our patients, particularly males, exhibited low self-esteem, sexual shame, and a tendency to postpone or avoid marriage. Among sexually active individuals, males reported significantly greater embarrassment when engaging in sexual activity, leading to a decreased frequency and avoidance of sexual encounters.

Healthcare providers often face challenges in initiating discussion or conducting examinations specifically focused on genital psoriasis [7, 12]. Similarly, patients often hesitate to discuss the condition with their physicians, with nearly half of the patients in a prior study reporting never having disclosed their genital psoriasis [12]. In our study, fewer than half of the respondents (40%) had ever been queried or examined by their doctors regarding genital psoriasis. In addition, 42.9% of the respondents had never raised their condition with their healthcare providers. Cather et al. reported that patients preferred being interviewed about genital psoriasis via telephone rather than face-to-face due to embarrassment or discomfort experienced by discussing such a sensitive subject [10]. We also investigated patients' perceptions of allowing a doctor to examine them. Surprisingly, males were significantly more amenable to allowing physicians to examine their genitalia.

Despite the profound impact of genital psoriasis on both sexual and overall well-being, a substantial proportion of patients continue to receive inadequate care from their healthcare providers [7, 8, 12]. The causative deficiency in communication may be attributed to the inherent sensitivity surrounding genital health and the challenges associated with initiating open discussion about issues involving genitalia.

Treatment of genital psoriasis presents a particular challenge due to the sensitive nature of genital skin and its response to medications. In addition, as many as two-thirds of patients with genital psoriasis have never sought formal treatment for their lesions [12]. Nonpharmacological interventions for genital psoriasis emphasize good hygiene and friction reduction. It is recommended to use mild, nonsoap cleansers to keep the genital area clean without causing irritation, and to wear loose-fitting clothing to avoid the Koebner phenomenon and irritation [8]. First-line therapies typically involve low-to-midpotency topical corticosteroids [5, 11]. However, potential side effects from prolonged corticosteroid use warrant consideration. Second-line treatments include calcineurin inhibitors, topical calcipotriol, and coal tar [8].

Topical calcineurin inhibitors (TCI) have demonstrated effectiveness and tolerability in managing both genital and facial psoriasis with minimal adverse effects. In a randomized, double-blinded, controlled study, topical tacrolimus achieved superior efficacy, with 67% of patients showing clear or excellent outcomes compared to 37% in the placebo group. In addition, there were no significant differences in symptoms such as burning, stinging, or itching [22]. Topical tacrolimus has also been found to be as effective as a midpotency topical corticosteroid [23].

Topical vitamin D analogs such as calcipotriol and calcitriol are another suggested long-term treatment for genital psoriasis. However, previous studies have demonstrated that they are less effective than topical corticosteroids, or TCI, and can develop more severe cutaneous irritation compared to TCI [24]. Coal tar's efficacy and safety have been reported in a study in which patients responded significantly to coal tar 2% and topical salicylate preparations twice daily at week 8 [25].

Crisaborole, a novel nonsteroidal phosphodiesterase-4 inhibitor, has shown promise in the management of genital psoriasis. It functions by increasing intracellular cyclic adenosine monophosphate levels, thereby reducing the production of proinflammatory cytokines such as Th1 (TNF-*α* and interferon-*γ*), Th2 (IL-4 and IL-13), and IL-17 [26, 27].

Roflumilast, another topical PDE-4 inhibitor, was studied in a phase IIb trial. Patients were randomized to receive either a placebo, roflumilast 0.3% cream, or roflumilast 0.15% cream. At week 12, 93% had an intertriginous area IGA score of 0 (indicating clear) in roflumilast 0.3% group, compared with 18% in the placebo group [28].

The International Psoriasis Council's new classification system for psoriasis severity recommends stratifying patients based on suitability for topical or systemic treatment, including those with facial or genital involvement. While systemic therapies are seldom prescribed for isolated genital psoriasis, they may be advantageous in severe cases involving genital lesions. In our study, methotrexate, cyclosporine, and acitretin were administered to patients with severe psoriasis complicated by genital involvement.

Ultraviolet phototherapy is generally not advised for genital psoriasis patients due to safety concerns [29]. Stern et al. [30] reported that prolonged exposure to photochemotherapy (psoralen plus ultraviolet A), as well as high doses of ultraviolet B exposure, were linked to an increased risk of genital tumors in males. However, one female patient in our study reported favorable outcomes with ultraviolet B phototherapy applied to both body and genital lesions. She underwent 27 sessions over 5 months, with a final dose of 0.86 J/cm^2^.

Biologic therapies, bolstered by promising results from new drugs, have shown the potential to improve the sexual dysfunction of psoriasis patients [21]. Ixekizumab, an anti-IL-17 agent, is presently the only biologic agent approved by the FDA as effective in treating genital psoriasis (grade of recommendation: B). In two double-blinded, placebo-controlled phase IIIb trials, ixekizumab demonstrated superior efficacy to placebo in treating moderate-to-severe genital psoriasis (body surface area ≥1%) and improving symptoms related to sexual activity [9, 31]. Other biologics, such as adalimumab and ustekinumab, have also been reported to be effective [32]. These findings highlight the potential of targeted therapies to mitigate the significant quality-of-life impairments associated with genital psoriasis. Early detection and intervention may further help prevent the functional and social sequelae of this debilitating condition. Our findings indicate that patients treated with anti-IL-17 agents (ixekizumab, secukinumab, and brodalumab) achieved mostly satisfactory outcomes, followed by those treated with an anti-IL-23 agent (guselkumab). In a recent randomized, controlled study, patients with genital psoriasis who took apremilast, an oral immunomodulating phosphodiesterase 4 inhibitor, showed statistically and clinically significant improvements in their genital Physician Global Assessment scores as well as improvements in their signs, symptoms, severity, and quality of life. [33].

A genital psoriasis awareness program by Meeuwis et al. [34] demonstrated symptom relief and improved quality of life with simple topical interventions over a short timeframe (6 weeks). These findings underscore the necessity of recognizing and prioritizing effective treatments to alleviate symptoms and improve quality of life.

Our study is limited by its small sample size and dependence on question-based data, which did not include physical examinations of all genital areas. Consequently, the information is subject to potential recall bias from the patients' self-reports.

## 5. Conclusions

Genital psoriasis has a significant incidence within the Thai population. Our study highlights the considerable psychosocial and psychosexual impact of genital psoriasis, particularly among male patients. Unfortunately, a potential gap in diagnostic and therapeutic attention for genital psoriasis was identified, with healthcare professionals sometimes overlooking the condition and patients underreporting their concerns. To optimize the management of psoriasis, healthcare providers must adopt a proactive stance in detecting and treating genital psoriasis, ensuring comprehensive care for patients.

## Figures and Tables

**Table 1:** Demographic profile of patients with genital psoriasis.

	Total (*n* = 50)
	*N* (%)
Sex	
Female	33 (66)
Male	17 (34)
Age (yr), mean ± SD	45.4 ± 13.4
Age at onset of psoriasis (yr), mean ± SD	31.6 ± 13.1
Age at onset of genital psoriasis (yr), mean ± SD	39.5 ± 12.2
BMI (kg/m^2^), mean ± SD	26 ± 5.2
Nationality	
Thai	49 (98)
Non-Thai (Chinese)	1 (2)
Status	
Married	25 (50)
Single	17 (34)
Divorced	5 (10)
Separated	3 (6)
Education (*N* = 48)	
Primary school	10 (20)
Secondary school	10 (20)
Bachelor's degree	22 (44)
Master's degree	6 (12)
History of smoking consumption	10 (20)
History of alcohol consumption	19 (38)
Psoriasis type	
Chronic plaque	45 (90)
Erythrodermic	5 (10)
PASI, median (IQR)	5.1 (1.7, 8.6)
BSA (%), median (IQR)	6 (2, 12.8)

BMI, body mass index; BSA, body surface area; PASI, psoriasis area severity index; SD, standard deviation; IQR, interquartile range.

**Table 2:** Clinical manifestations of genital psoriasis.

	Total (*N* = 50)
	*N* (%)
Currently has genital involvement	
Yes	17 (34)
No	33 (66)
Genital involvement was the first area of psoriasis	4 (8)
History of flexural involvement of psoriasis	
Inframammary	44 (88)
Perianal	33 (66)
Inguinal	30 (60)
Axilla	30 (60)
Genital involvement areas	
Male (*n* = 17)	
Shaft of penis	9 (52.9)
Glans penis	8 (47.1)
Scrotum	5 (29.4)
Female (*n* = 33)	
Mon pubis	16 (48.5)
Labia majora	6 (18.2)
Perineum	5 (15.2)
Symptoms	
Itch	41 (82)
Burning	10 (20)
Pain	10 (20)
Dyspareunia	2 (4)
Related areas of involvement (male and female)	
Pubic area	22 (44)
Buttocks	17 (34)
Inguinal area	16 (32)
Intergluteal cleft	12 (24)
Sacrum area	10 (20)
Perianal area	9 (18)
Intergluteal fold	3 (6)
Aggravating factors of genital psoriasis	
Stress	29 (58)
Sleep deprivation	23 (46)
Illness	15 (30)
Scratching	15 (30)
Wearing tight-fitting clothing	9 (18)
Alcohol consumption	8 (16)
Menstruation	6 (12)
Sexual intercourse	1 (2)
Cold weather	1 (2)
Treatment discontinuation	1 (2)

**Table 3:** Quality of life, sexual health, and perception of patients with genital psoriasis.

	Total (*N* = 50) *N* (%)	Female (*N* = 33) *N* (%)	Male (*N* = 17) *N* (%)	*P*	OR_M_ (95% CI)
	Median (P25, P75)	Median (P25, P75)	Median (P25, P75)		
Quality of life					
Symptoms					
Itch	41 (82)	29 (87.9)	12 (70.6)	0.242	
Burning	10 (20)	6 (18.2)	4 (23.5)	0.717	
Pain	10 (20)	6 (18.2)	4 (23.5)	0.717	
Dyspareunia	2 (4)	0 (0)	2 (11.8)	0.111	
DLQI (*N* = 49)		(*N* = 32)	(*N* = 17)		
	7 (3, 14)	6.5 (3, 10)	10 (3, 16)	0.328	
DLQI no. 9 (*N* = 48)		(*N* = 31)	(*N* = 17)	0.016	4.7 (1.3, 17.3)
Answer 0 (not at all)	33 (68.8)	25(80.6)	8 (47.1)		
Answer 1–3 (a little, a lot, and very much)	15 (31.3)	6 (19.4)	9 (52.9)		
Low self-esteem	27 (54)	14 (42.4)	13 (76.5)	0.022	4.4 (1.2, 16.4)
Marital choice (*N* = 49)	13 (26.5)	5 (15.6)	8 (47.1)	0.038	4.8 (1.2, 18.5)
Underwear choice					
Loose (*N* = 26)	20 (76.9)	15 (78.9)	5 (71.4)	1.000	
None (*N* = 24)	5 (20.8)	2 (11.1)	3 (50)	0.078	
Tight (*N* = 24)	2 (8.3)	2 (11.1)	0 (0)	1.000	
Sexual health					
Sexually active (*N* = 25)		*N* = 14	*N* = 11		10.5 (1.5, 72.8)
Avoidance of SI	12 (48)	5 (35.7)	7 (63.6)	0.165	
Embarrassed by SI	9 (36)	2 (14.3)	7 (63.6)	0.017	
Physicians' and patients' perceptions of genital psoriasis					
Patients provided physicians with information (*N* = 49)					
Yes	28 (57.1)	20 (62.5)	8 (47.1)	0.299	
No	21 (42.9)	12 (37.5)	9 (52.9)		
Physicians asked/examined					
Yes	20 (40)	11 (33.3)	9 (52.9)	0.180	
No	30 (60)	22 (66.7)	8 (47.1)		
Patient consented to being examined by a physician	25 (50)	13 (39.4)	12 (70.6)	0.037	3.7 (1, 12.9)

DLQI, dermatology life quality index; OR_M_, odds ratio of males compared to females; SI; sexual intercourse.

**Table 4:** Comparison of patients with psoriasis with active or inactive genital psoriasis.

	Total (*N* = 50) N (%)	Previous (*N* = 33) N (%)	Current (*N* = 17) N (%)	*P*	OR
	Median (P25, P75)	Median (P25, P75)	Median (P25, P75)		
PASI	5 (1.7, 8.6)	4.2 (1.3, 7.8)	7.9 (2.9, 12.6)	0.041	
BSA (%)	6 (2, 12.8)	5 (1, 10)	10 (3, 15)	0.082	
DLQI (*N* = 49)	7 (3, 14)	6 (3, 10.8)	9 (3.5, 17)	0.218	
Pubic area	22 (44)	10 (30.3)	12 (70.6)	0.007	5.5 (1.5, 19.9)
Intergluteal cleft	12 (24)	5 (15.2)	7 (41.2)	0.077	
Low self-esteem	27 (54)	16 (48.5)	11 (64.7)	0.276	
Underwear choice	27 (54)	18 (54.5)	9 (52.9)	0.914	
		(*N* = 32)	(*N* = 17)		
Marital choice (*N* = 49)	13 (26.5)	7 (21.9)	6 (35.3)	0.331	
Sexual health					
Sexually active (*N* = 25)		*N* = 19	*N* = 6		
Avoidance of SI	12 (48)	8 (42.1)	4 (66.7)	0.281	
Embarrassed by SI	9 (36)	7 (36.8)	2 (33.3)	0.637	

BSA, body surface area; DLQI, dermatology life quality index; OR, odds ratio; PASI, psoriasis area severity index; SI, sexual intercourse.

**Table 5:** Treatment of patients with genital psoriasis.

Currently being treated for genital psoriasis	Total (*N* = 17)
	*N* (%)
Yes	14 (82.4)
No	3 (17.6)

Current treatment for genital and other psoriatic lesions	Total (*N* = 14)
	*N* (%)

Emollient	14 (100)
Topical treatment	13 (92.9)
Conventional systemic treatment	6 (42.9)
Methotrexate	4 (28.6)
Acitretin	1 (7.1)
Cyclosporine	1 (7.1)
Biologic (secukinumab)	2 (14.3)
Phototherapy (NBUVB)	1 (7.1)
Antihistamine (loratadine)	1 (7.1)

Previous and current treatments that patients perceived as improving their genital psoriasis	Total (*N* = 50)
	*N* (%)

Topical treatment	29 (58)
Emollient	18 (36)
Conventional systemic treatment	15 (30)
Methotrexate	7 (14)
Cyclosporine	5 (10)
Acitretin	3 (6)
Biologic	7 (14)
Ixekizumab	3 (6)
Secukinumab	2 (4)
Brodalumab	1 (2)
Guselkumab	1 (2)
Phototherapy (NBUVB)	1 (2)

NBUVB, narrowband ultraviolet B phototherapy.

## Data Availability

The data that support the findings of this study are available from the corresponding author upon reasonable request.
